# Engineered Picornavirus VPg-RNA Substrates: Analysis of a Tyrosyl-RNA Phosphodiesterase Activity

**DOI:** 10.1371/journal.pone.0016559

**Published:** 2011-03-07

**Authors:** Janet M. Rozovics, Richard Virgen-Slane, Bert L. Semler

**Affiliations:** Department of Microbiology and Molecular Genetics, School of Medicine, University of California Irvine, Irvine, California, United States of America; Nanyang Technological University, Singapore

## Abstract

Using poliovirus, the prototypic member of *Picornaviridae*, we have further characterized a host cell enzymatic activity found in uninfected cells, termed “unlinkase,” that recognizes and cleaves the unique 5′ tyrosyl-RNA phosphodiester bond found at the 5′ end of picornavirus virion RNAs. This bond connects VPg, a viral-encoded protein primer essential for RNA replication, to the viral RNA; it is cleaved from virion RNA prior to its engaging in protein synthesis as mRNA. Due to VPg retention on nascent RNA strands and replication templates, but not on viral mRNA, we hypothesize that picornaviruses utilize unlinkase activity as a means of controlling the ratio of viral RNAs that are translated versus those that either serve as RNA replication templates or are encapsidated. To test our hypothesis and further characterize this enzyme, we have developed a novel assay to detect unlinkase activity. We demonstrate that unlinkase activity can be detected using this assay, that this unique activity remains unchanged over the course of a poliovirus infection in HeLa cells, and that unlinkase activity is unaffected by the presence of exogenous VPg or anti-VPg antibodies. Furthermore, we have determined that unlinkase recognizes and cleaves a human rhinovirus-poliovirus chimeric substrate with the same efficiency as the poliovirus substrate.

## Introduction

The presence of protein-nucleic acid covalent bonds is not unusual in viral pathogens. Multiple virus families have a protein covalently attached to their genomes; some examples include *Potyviridae*, *Adenoviridae, Nepoviridae,* and *Picornaviridae* [for review, see [1], [2]]. In the case of picornaviruses this viral protein, termed VPg (Viral Protein genome-linked), is located at the 5′ end of all nascent picornavirus genomes as a result of serving as a primer for RNA synthesis [3], [4], [5], [6], [7], [8], [9], [10], [11].

**Figure 1:**
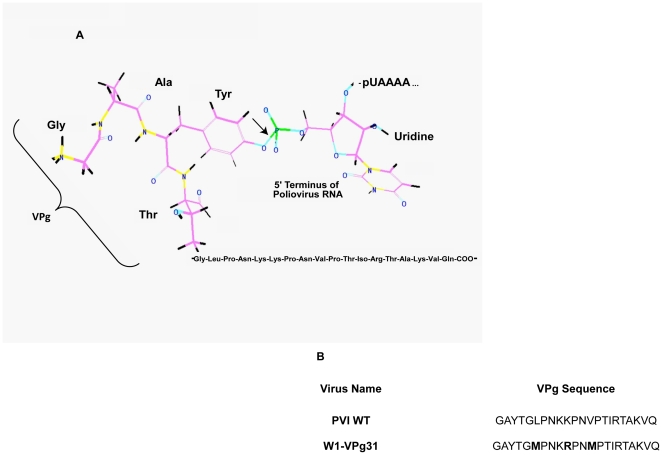
VPg-RNA covalent linkage and VPg sequences of wild type and mutated W1-VPg31 polioviruses. Schematic of the VPg-RNA covalent linkage between the single tyrosine residue in poliovirus VPg and the first uridine at the 5′ terminus of the genomic RNA (A). Image was generated using ChemDraw software. The arrow indicates where unlinkase cleaves the bond. (B) VPg sequences of wild type poliovirus and W1-VPg31 (wild type poliovirus mutated to encode two methionines in the VPg coding region).

The occurrence of protein-nucleic acid covalent bonds in uninfected cells is slightly more novel. These linkages result from the failure of a topoisomerase to dissociate properly from target DNA [for review see [12]]. In such instances additional enzymes, termed tyrosyl-DNA phosphodiesterases, are required to resolve the phosphodiester bond formed with the nucleic acid. Currently, there are two documented examples of these enzymes in eukaryotic cells: tyrosyl-DNA phosphodiesterase 1 and tyrosyl-DNA phosphodiesterase 2 (formerly TTRAP), which primarily recognize and cleave 3′ and 5′ tyrosyl-DNA phosphodiester bonds, respectively [13], [14], [15], [16]. Interestingly, there is evidence for a tyrosyl-RNA phosphodiesterase activity in eukaryotic cells. Existence of this enzyme is demonstrated by the efficient removal of the picornavirus protein VPg, which is attached to the genomic RNA via an O^4^-(5′-Uridylyl)tyrosine bond, from the 5′ end of the viral genome (Figure 1) [17], [18]. Although this activity has been given several names in the literature, including unlinking enzyme [18], VPg unlinkase [19], and uridylylpolynucleotide-(5′ P->O)-tyrosine phosphodiesterase (Y-pUpN PDE) [20], in this manuscript we will refer to the enzymatic activity as “unlinkase.”

The identity of unlinkase is unknown, but the activity has been partially characterized. Activity has been reported in wheat germ extracts [17], mouse ascites Krebs II cells [21], rabbit reticulocyte lysate [22], [23], [24], and in the nucleus and cytoplasm of HeLa cells [17]. Unlinkase has the hallmarks of a bonafide enzyme, since the activity is dependent on Mg^2+^ or Mn^2+^ [17], [22] and is inhibited in the presence of vanadate, SDS, Zn^2+^ and EDTA [22], [24]. Reducing agents, translation inhibitors, RNase, and protease inhibitors do not appear to affect unlinkase activity [22]. It is unknown whether unlinkase activity results from a single enzyme, a complex of hetero- or homo-multimers, or an RNP complex with the viral RNA. Partially purified preparations of the enzyme have yielded low turnover numbers, suggesting that the purified protein was only a component of a potential complex or that the enzyme responsible possesses a very different function in the uninfected cell and cleaving of the tyrosyl-RNA bond in picornavirus genomic RNA is simply a minor role of the enzyme [18]. It has also been demonstrated that the length of the attached RNA, not the integrity of the VPg, is more important for efficient unlinkase cleavage [18], [19]. Unlinkase activity to date has been described to recognize and cleave the VPg from the genomic RNA of enteroviruses [17], [18], cardioviruses [25], and aphthoviruses [22]. The activity is very specific for a tyrosyl-RNA phosphodiester bond, as a tyrosyl-DNA bond is not cleaved in the presence of unlinkase [unpublished data, [20]], nor is the serine-RNA bond of cowpea mosaic virus, a member of the *Comovirinae* virus subfamily [21], [26].

A key unresolved issue is the role of VPg cleavage from virion RNA by unlinkase in the replication cycle of picornaviruses. VPg is cleaved from picornaviral RNA genomes that are destined to serve as templates for translation; the viral protein is retained on templates for replication as well as genomes that are encapsidated [8], [27], [28], [29], [30], [31]. VPg, however, is not cleaved from VPg-pU and VPg-pUpU substrates (which normally serve as primers for replication during infection) [18], [19]. We hypothesize that unlinkase activity is utilized by picornaviruses to distinguish templates for translation from those that are to serve as templates for replication or that are encapsidated in virions for further rounds of infection. This hypothesis would also support the possibility that VPg has a role in encapsidation of the genome.

To test the hypothesis that unlinkase activity is required to differentiate templates for translation from those to be used in replication, the enzyme responsible for the activity must be purified and identified. Despite intensive efforts from our lab and other research groups, this is still a work in progress. However, while the enzyme has not been identified, further characterization of the activity has been accomplished. We have developed a novel assay that visualizes migration of a ^35^S-methionine radiolabeled VPg-RNA substrate generated *in vivo* to determine if unlinkase activity is present. Here, we report that unlinkase activity can be detected using this assay and confirm previous reports that the full-length virion RNA is a much more efficient substrate than a VPg-nonanucleotide substrate. We also demonstrate that this unique activity remains unchanged over the course of a poliovirus infection in HeLa cells and that it is unaffected by the presence of exogenous VPg or anti-VPg antibodies. Furthermore, we have determined that unlinkase recognizes and cleaves a human rhinovirus-poliovirus chimeric substrate with the same efficiency as the poliovirus substrate.

## Materials and Methods

### Extracts, partially-purified unlinkase fractions, synthetic poliovirus VPg, and antibodies

Cytoplasmic extract for unlinkase analysis was prepared from HeLa, NGP, SK-OV-3 and K562 cell lines. HeLa cells [32], [33] were grown in suspension culture in SMEM supplemented with 8% newborn calf serum (NCS), K562 cells [34] were grown in DMEM supplemented with 10% fetal bovine serum (FBS), NGP cells [35], [36] were grown in DMEM supplemented with 20% FBS. SK-OV-3 cell monolayers (American Type Culture Collection number: HTB-77) were grown in RPMI media supplemented with 12% FBS. Cells were pelleted, washed with 1X PBS, and subjected to an S10 cytoplasmic preparation as described elsewhere [37]. However, extracts used for further unlinkase purification were not subjected to micrococcal nuclease treatment, but were instead used to prepare a ribosomal salt wash (RSW) fraction. To prepare the RSW, HeLa cell S10 cytoplasmic extract was centrifuged at a max RCF of 370,500×g for one hour at 4°C. The supernatant was discarded and the pellet was resuspended in 1X hypotonic buffer (20 mM HEPES, pH 7.4, 10 mM MgOAc, 1 mM DTT) and adjusted to an OD_260 nm_ of 240–480 units/ml (1 µl of sample resuspended in 400 µl of 1X hypotonic buffer equals OD_260 nm_ units/ml). KCl was added to a final concentration of 0.5 M and the mixture was incubated at 4°C for 15 minutes. The resuspended pellet was then centrifuged again at a max RCF of 370,500×g for one hour at 4°C. The supernatant was collected and dialyzed into unlinkase buffer (20 mM Tris-Cl, pH 7.5, 1 mM DTT, 5% glycerol). Dialyzed fractions were stored at −80°C. Micrococcal nuclease-treated rabbit reticulocyte lysate was obtained from Promega.

To obtain cytoplasmic extracts from infected cells, HeLa cells in suspension culture were pelleted and washed twice with 1X PBS. Pelleted cells were resuspended in half of the required total volume of serum-free SMEM and infected with wild type poliovirus at a multiplicity of infection (MOI) of 20; adsorption took place at room temperature for 30 minutes. After adsorption, 8% NCS, remaining media and HEPES, pH 7.4 (final concentration 20 mM) were added; the infection was carried out at 37°C. S10 cytoplasmic extracts generated from poliovirus-infected HeLa cells at zero, two, four and six hours post-infection, as well as a zero and six hour time point from mock-infected HeLa cells, were prepared as described [37], with the exception that they were not subjected to treatment with microccocal nuclease.

To generate partially-purified, enriched unlinkase activity fractions via cation-and-anion exchange chromatography (Fraction CA), RSW was subjected to cation-exchange chromatography (SP Sepharose FF, GE Healthcare), and proteins were eluted from the column using increasing concentrations of NaCl in unlinkase buffer. Protein fractions were dialyzed against unlinkase buffer and analyzed for unlinkase activity. Peak fractions were pooled, subjected to anion-exchange chromatography (AEC) (DEAE Sepharose FF, GE Healthcare), and proteins were eluted using increasing concentrations of NaCl in unlinkase buffer. Protein fractions were dialyzed into unlinkase buffer and analyzed for unlinkase activity; fractions containing peak activity were then used in further assays.

To generate partially-purified, enriched unlinkase activity fractions using sucrose gradient fractionation and anion-exchange chromatography (Fraction SA), RSW was initially applied to one of two gradients: 7%–47% sucrose w/w (20 mM Tris pH 7.5, 5 mM MgCl_2_, 100 mM KCl) or 15–30% sucrose w/w (20 mM Tris pH 7.5, 5 mM MgCl_2_, 100 mM KCl). Gradient centrifugation was carried out in a Beckman SW41 rotor at 32,000 rpm for three hours at 4°C. Fractions were collected, dialyzed into unlinkase buffer, and analyzed for unlinkase activity. Fractions exhibiting the highest levels of unlinkase activity from sucrose gradients were pooled and subjected to anion-exchange chromatography (DEAE Sepharose FF, GE Healthcare), and proteins were eluted using increasing concentrations of NaCl in unlinkase buffer. Protein fractions were dialyzed into unlinkase buffer and analyzed for unlinkase activity; fractions containing peak activity were then used in further assays.

Synthetic poliovirus VPg peptide was produced by BioBlocks, San Diego, CA. This synthetic peptide was used to generate anti-VPg polyclonal antibodies in rabbits (Bethyl Laboratories).

### Cloning, transcription, and transfection of the human rhinovirus 14-poliovirus S1-VPgR1 chimera

The site selection for the methionine mutations of the VPg coding region of HRV14 was based on previously published work [38], [39], [40]. The poliovirus cDNA plasmid, pT7PV1, was mutated (Table 1: oligonucleotides 1–4) to flank the VPg coding sequence with PpuMI and ApaI restriction sites. The native poliovirus VPg coding region was excised using the engineered PpuMI and ApaI restriction sites, replaced with the HRV14 VPg M[6], [17] cartridge, which consisted of two pairs of overlapping, complementary oligonucleotides (Table 1: oligonucleotides 5–8) and cloned into the gel-purified, linear template in a single ligation reaction. The sequence of the final product, pT7-S1-VPgR1, was verified via DNA sequencing using oligonucleotide 9 (Table 1).

**Table 1:** Oligonucleotides used for cloning of S1-VPgR1.

Oligonucleotide #	Name	Oligonucleotide Sequence
**1**	**pT7-PV1_PpuMI(+)**	**5′-TTTGCTGGACACCAGGGACCCTACACTGGTTTACCAAAC-3′**
**2**	**pT7-PV1_PpuMI(-)**	**5′-GTTTGGTAAACCAGTGTAGGGTCCCTGGTGTCCAGCAAA-3′**
**3**	**pT7-PV1_ApaI(+)**	**5′-CAGCAAAGGTACAAGGGCCCGGGTTCGATTACGCAG-3′**
**4**	**pT7-PV1_ApaI(-)**	**5′-CTGCGTAATCGAACCCGGGCCCTTGTACCTTTGCTG-3′**
**5**	**HRV14_P1(+)**	**5′-GACCCTATTCTGGTATGCCGCCTCACAATAAACTAA-3′**
**6**	**HRV14_P1(-)**	**5′-GCTTTTAGTTTATTGTGAGGCGGCATACCAGAATAGG-3′**
**7**	**HRV14_P2(+)**	**5′-AAGCCCCAACTATGCGCCCAGTTGTTGTGCAAGGGCC-3′**
**8**	**HRV14_P2(-)**	**5′-CTTGCACAACAACTGGGCGCATAGTTGGG-3′**
**9**	**3C_5628(-)**	**5′-TCAAGATTGGTTCCTGCTTGAT-3′**
**10**	**JT_5060(+)**	**5′-GAGAGAAACAGAAGATCCAA-3′**

The pT7-S1-VPgR1 template was linearized with EcoRI, phenol/chloroform extracted, ethanol precipitated and transcribed *in vitro*. Serial dilutions of the transcription reaction were incubated with 1 mg/ml DEAE-dextran in TS buffer (19 mM Tris-HCl, 4.4 mM KCl, 0.4 mM Na_2_HPO_4_, 137 mM NaCl, 490 mM MgCl_2_, 0.7 mM CaCl_2_, adjusted to pH 7.4) for 30 minutes at room temperature, and 250 µl of these mixtures were used to transfect HeLa cell monolayers in 60 mm plates for 30 minutes at room temperature. Following transfection, the monolayers were overlaid with DMEM supplemented with 10% FBS and 0.45% agarose and incubated at 37°C. Sixty hours post-transfection, plaques were isolated and used to infect HeLa cell monolayers to generate viral stocks. Viral titers were determined by plaque assay. The viral stocks were passaged three times, and the P4 stock was used to generate the purified unlinkase substrate. To verify that the VPg mutation was retained after transfection and passaging of the mutant S1-VPgR1 virus, HeLa cell monolayers were infected at an MOI of 25 for 5.5 hours and total cellular RNA was extracted using TRI Reagent (Molecular Research Center, Inc.) and subjected to RT-PCR (Table 1: oligonucleotide 9) to produce a viral cDNA fragment. The cDNA fragment was amplified by PCR (Table 1: oligonucleotides 9 and 10) and then subjected to DNA sequencing.

### Preparation of picornavirus virion RNA substrates

The ^35^S-methionine-labeled virion RNA from the W1-VPg31 [38], [39] and S1-VPgR1 viruses were prepared as follows: HeLa cells in suspension culture were prepared for infection as described above, with the exception of using methionine-free DMEM (MP Biomedicals). The infected HeLa cells were starved of methionine for two hours at 37°C after adsorption and then 1 mCi of ^35^S-methionine was added. At six hours post-infection, cells were pelleted and resuspended in RSB+Mg^2+^ buffer (0.01 M NaCl, 0.01 M Tris-Cl, pH 7.5, 1.5 mM MgCl_2_). The resuspended pellet was freeze-thawed five times and centrifuged again to pellet nuclei and cellular debris. The supernatant was collected and the pellet was washed again in RSB+Mg^2+^ buffer, centrifuged, and the resulting supernatant was pooled with the original supernatant. The supernatant was centrifuged in a Beckman Ti 70 rotor at 24°C at 29,700 rpm for 3.5 hours to pellet virions. The resulting supernatant was discarded, the pellet was resuspended in 0.1 buffer (0.1 M NaCl, 10 mM Tris-Cl, pH 7.5, 5 mM EDTA, 0.5% SDS), applied to a 15–30% sucrose gradient (5 mM EDTA, 0.1 M NaCl, 10 mM Tris-HCl, pH 7.5, 0.5% SDS), and centrifuged at 27,700 rpm in a Beckman SW41 rotor for 2.5 hours at 24°C. Fractions of the gradient were collected and the radioactivity of each fraction was determined by scintillation counting; peak fractions were pooled and subjected to two rounds of phenol/chloroform extraction and ethanol precipitation. The final volume of the virion RNA prep was adjusted to 700 cpm/µl (∼0.15 pmol/µl).

The VPg-nonanucleotide substrate was generated by digesting the full-length substrate with RNase T1 (200 units) for one hour at 37°C in TM Buffer (30 mM Tris-Cl, pH 7.5, 10 mM MgCl_2_). This mixture was then directly added to an unlinkase source or RNase A (Sigma) in assays.

### Single-cycle viral growth analysis

Monolayers of HeLa cells [41] were washed twice with 1X PBS and infected with either poliovirus or S1-VPgR1 virus at a multiplicity of infection (MOI) of 20. Following a 30 minute adsorption period at room temperature, cells were washed three times with 1X PBS to remove unadsorbed virus and then overlaid with DMEM supplemented with 10% FBS. Infection was carried out at 37°C. At zero, two, four, and six hours post-infection, the cells were scraped and collected with media. Cell suspensions were subjected to five freeze–thaw cycles and total plaque-forming units were determined via plaque assay.

### Analysis of unlinkase activity and competition assays

For each experiment, the equivalent of 700 cpm (∼0.15 pmol)/reaction of radiolabeled substrate was used. VPg from the poliovirus-rhinovirus chimera was differentially labeled compared to W1-VPg31; to compensate, specific activities of the viral RNA preparations were matched via addition of purified unlabeled W1-VPg31 virion RNA to the radiolabeled W1-VPg31 virion RNA. The final physical amounts of RNA for each reaction were equal. Unless noted, the substrate was then incubated with 20 µg of protein from a source of unlinkase in the presence of 2 mM MgCl_2_ for 30 minutes. Reaction mixtures from either full-length or VPg-nonanucleotide substrate experiments were resolved via 13.5% Tris-tricine PAGE analysis. Gels were dried immediately after electrophoresis. Following autoradiography using a phosphorimager screen, results were analyzed using Quantity One software (Bio-Rad).

For competition assays, 0.1, 0.5, or 1.0 µg of exogenous, synthetic poliovirus VPg was pre-incubated with 20 µg of protein from RSW (as an unlinkase source) for 15 minutes at 30°C before the addition of ^35^S-methionine-labeled W1-VPg31 virion RNA substrate; the reaction was incubated for an additional 10 minutes at 30°C before the products were subjected to 13.5% Tris-tricine PAGE analysis as previously described.

## Results

### Optimal assay conditions for detecting unlinkase activity using full-length poliovirus virion RNA ^35^S-methionine-labeled substrate

We chose the approach of radiolabeling the mutant poliovirus W1-VPg31 (Figure 1B) to clearly visualize liberation of VPg from the VPg-RNA substrate. This approach also allows for inspection of the cleaved VPg, as crude preparations of unlinkase from cellular extracts contain a protease that degrades free VPg [24]. A previously described method of detecting unlinkase activity via measuring the partition of liberated RNA to the phenol phase is problematic due to the potential degradation of VPg; the products that result from proteolysis partition to the aqueous phase, possibly leading to ambiguous interpretations of the data [22], [24].

**Figure 2:**
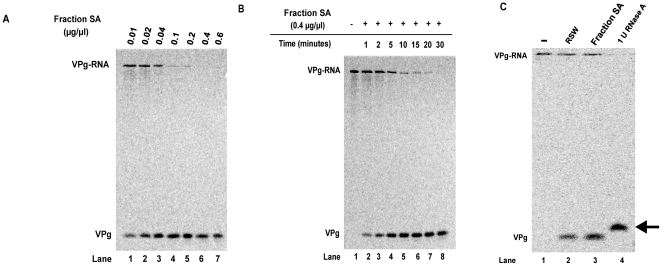
Optimal assay conditions for detecting unlinkase activity using full-length poliovirus virion RNA ^35^S-methionine labeled substrate. A protein chromatography fraction enriched for unlinkase activity was incubated with full-length ^35^S-methionine radiolabeled W1-VPg 31 virion RNA substrate with either (A) increasing amounts of protein (0.01, 0.02, 0.04, 0.1, 0.2, 0.4, and 0.6 µg/µl) from an enriched source of unlinkase activity (Fraction SA) at 30°C for 30 minutes or (B) increasing incubation time (0, 1, 2, 5, 10, 15, 20, and 30 minutes) with 0.4 µg/µl of protein from a partially-purified fraction of unlinkase activity (Fraction SA) to determine optimal assay conditions for the full-length substrate. (C) ^35^S-methionine radiolabeled W1-VPg 31 virion RNA substrate was mock-incubated (lane 1), incubated with 0.8 µg/µl RSW (lane 2), 0.4 µg/µl of protein from a partially-purified fraction of unlinkase activity (Fraction SA) (lane 3), or one unit of RNase A (lane 4) to differentiate between non-specific nuclease activity and authentic unlinkase activity.

To determine optimal conditions for complete digestion of the picornavirus substrate using our novel assay, we tested different protein concentrations of an enriched unlinkase activity source along with different reaction incubation times. Increasing concentrations of an unlinkase-enriched protein fraction (Fraction SA) were incubated with radiolabeled W1-VPg31 substrate (Figure 2A), and we determined that 0.4 µg/µl, or 20 µg total, was the optimal concentration for complete digestion of the full-length poliovirus substrate (Figure 2A, lane 6) when the reaction was allowed to incubate at 30°C for 30 minutes. To confirm that 30 minutes was the appropriate incubation time for unlinkase activity on the radiolabeled substrate, a time-course assay using a concentration of 0.4 µg/µl was carried out (Figure 2B). This assay confirmed that 30 minutes was optimal for complete cleavage of the substrate (Figure 2B, lane 8). Therefore, a concentration of 0.4 µg/µl of a partially-purified source of unlinkase incubated for 30 minutes at 30°C is sufficient to achieve complete cleavage of the radiolabeled substrate.

To rule out the possibility that the unlinkase activity we observed in our assay was the result of a non-specific nuclease, we incubated our poliovirus substrate with RNase A (a single-strand nuclease) and compared the migration of the resulting radiolabeled VPg product to the VPg product that resulted when our substrate was incubated with a source of unlinkase activity. W1-VPg31 radiolabeled substrate was incubated with RSW (Figure 2C, lane 2), protein from a partially-purified unlinkase fraction (Fraction SA) (Figure 2C, lane 3), or RNase A (Figure 2C, lane 4). Treatment with RNase A would be predicted to produce a VPg-pUp product because RNase cannot remove the final attached nucleotide from the labeled VPg. Consistent with this prediction, the resulting product (denoted by the arrow) forms a band that migrates with a slightly slower electrophoretic mobility than the product of radiolabeled substrate treated with a source of unlinkase (Figure 2C, compare lane 4 to lanes 2 and 3). Additionally, significant levels of intact virion RNA are present when incubated with highly enriched fractions of unlinkase activity, further indicating that our VPg cleavage results are not due to a non-specific nuclease (data not shown).

### Full-length poliovirus genomic RNA is a more efficient substrate than virion RNA treated with RNase T1

**Figure 3:**
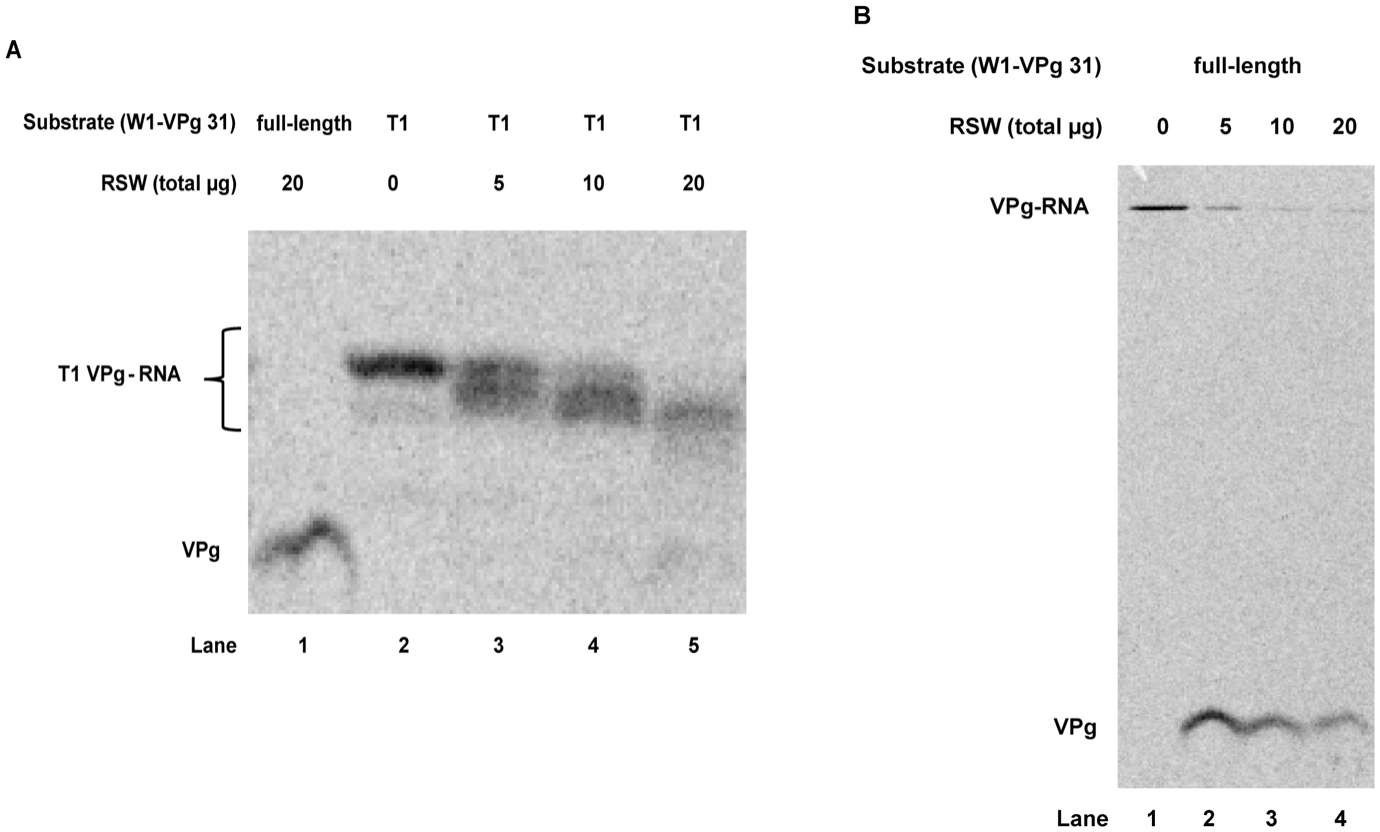
Efficiency comparison of truncated versus full-length ^35^S-methionine labeled substrate. ^35^S-methionine radiolabeled W1-VPg 31 substrate that was treated with RNase T1 (A) or untreated (B) was incubated with increasing amounts of total protein from RSW (0, 5, 10, and 20 µg) as a source of unlinkase activity to demonstrate the efficiency of the truncated versus the full-length substrate.

Other investigators have demonstrated that VPg attached to the first nine nucleotides of the poliovirus genome, generated by treatment of the full-length substrate with RNase T1, is a suitable, if inefficient, substrate for unlinkase activity [18], [19]. In our initial studies, we utilized this VPg-nonanucleotide substrate to determine the presence and levels of unlinkase activity. Our rationale for using this substrate was to visualize levels of liberated VPg versus full-length VPg-RNA substrate in our Tris-tricine polyacrylamide gels, as full-length virion RNA should not be able to enter into such a matrix. However, when compared to full-length, we determined that the efficiency of VPg removal from the truncated VPg-nonanucleotide substrate was noticeably reduced (Figure 3A, compare lanes 1 and 5) using RSW as a source of unlinkase activity. When treated with RNase T1, full-length poliovirus virion RNA was observed to generate three labeled products, with the slowest-migrating product being the most prominent (Figure 3A, lane 2). It is likely that this larger product is the VPg-nonanucleotide substrate and the less prominent bands are the result of over-digestion of RNase T1, which primarily recognizes and cleaves 3′ single-stranded guanidine residues, but can also cleave single-stranded adenosine residues if RNase T1 is present in excess amounts. Interestingly, cleavage of this truncated substrate is incomplete in the presence of saturating amounts of RSW (Figure 3A, compare lanes 1 and 5). In fact, an intermediate is observed (Figure 3A, lanes 3–5). By comparison (Figure 3B), full-length poliovirus substrate that has not been pre-treated with RNase T1 has the VPg efficiently cleaved from the virion RNA in the presence of unlinkase; no intermediate products are observed (Figure 3B, lanes 2–4). We also observed uncleaved full-length virion RNA substrate (Figure 3B), although it was trapped at the top of the separating gel and could not enter the matrix. Additionally, at higher concentrations of RSW (Figure 3A, lane 5 and Figure 3B, lane 4), more free VPg was observed to be degraded by an unidentified cellular protease [24]; we observe reduced proteolytic degradation of liberated VPg as unlinkase activity is further purified (data not shown). Based on these data, we conclude that not only is full-length poliovirus virion RNA a more efficient substrate for unlinkase activity than the VPg-nonanucleotide substrate, but the shorter substrate forms an intermediate that cannot be cleaved by unlinkase, confirming that the length of the RNA plays a role in the activity of this enzyme.

### Unlinkase activity remains unchanged during poliovirus infection of HeLa cells

**Figure 4:**
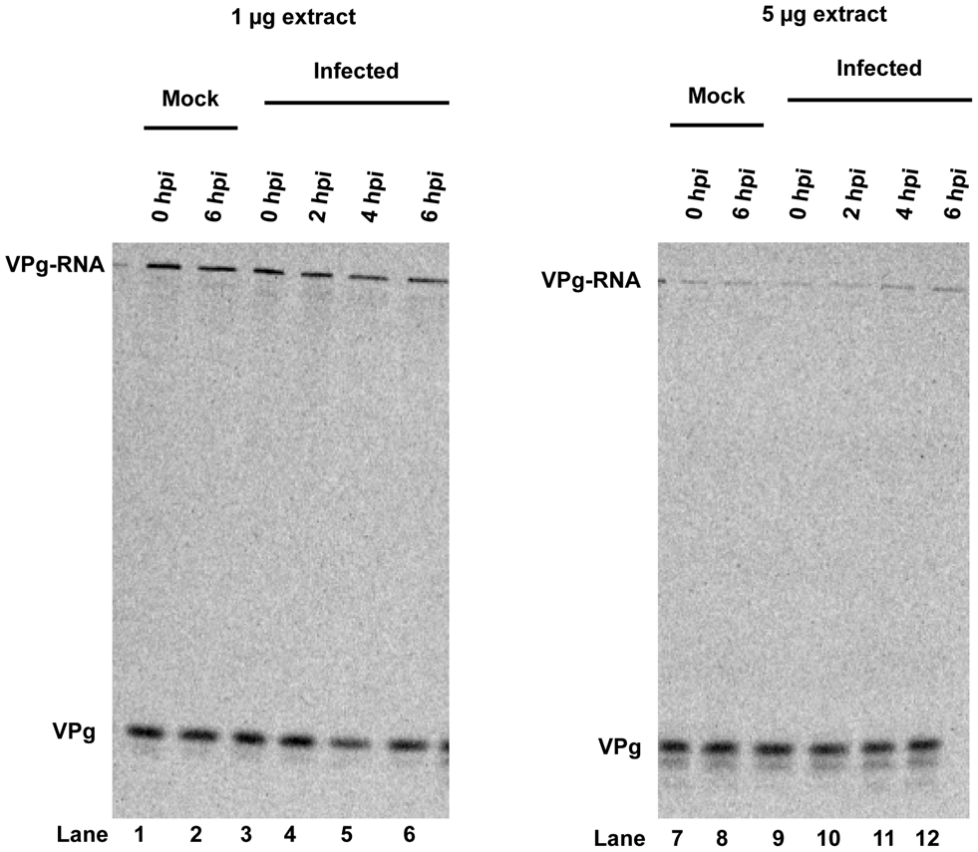
Unlinkase activity remains unchanged during poliovirus infection. HeLa cells were infected with wild type poliovirus and cytoplasmic extracts were collected at 0 (lanes 3 and 9), 2 (lanes 4 and 10), 4 (lanes 5 and 11), or 6 (lanes 6 and 12) hours post-infection. 1 µg (lanes 1–6) or 5 µg (lanes 7–12) of total protein from these extracts was incubated with full-length ^35^S-methionine radiolabeled W1-VPg 31 virion RNA substrate. No difference in activity was observed between the mock lanes (lanes 1, 2, 7, and 8) and the different time points collected during the infection.

Although it has been reported that unlinkase activity was not altered at late times after poliovirus infection [17], [19], it was not clear at what time during the infection unlinkase activity was assayed or if a more sensitive assay could detect minor differences in activity over a typical picornavirus infection. To address these unresolved questions, we carried out a wild type poliovirus infection of HeLa cells and generated cytoplasmic extracts from infected cells at zero, two, four, or six hours post infection. These extracts were then assayed for unlinkase activity (Figure 4). When compared to the mock-infected cells harvested at zero and six hours post infection (Figure 4, lanes 1, 2, 7, and 8), no measurable difference in unlinkase activity was observed among the different time points when 1 µg (Figure 4, lanes 3–6) or 5 µg (Figure 4, lanes 9–12) of total protein from the extract was analyzed. We conclude that unlinkase activity remains unchanged over the time course of a poliovirus infection, at least under the conditions we employed.

### Extracts from different human cell lines have varying levels of unlinkase activity

**Figure 5:**
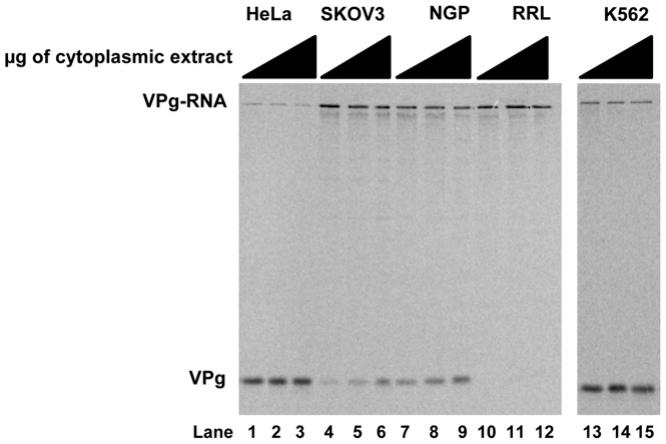
Unlinkase activity in different cytoplasmic extracts from human cell lines. Increasing amounts of total protein (0.1 µg/µl, lanes 1, 4, 7, 10, and 13; 0.2 µg/µl, lanes 2, 5, 8, 11, and 14; 0.4 µg/µl, lanes 3, 6, 9, 12, and 15) from cytoplasmic extracts from different cell lines were incubated with ^35^S-methionine radiolabeled W1-VPg 31 virion RNA substrate. HeLa (lanes 1–3), SK-OV-3 (lanes 4–6), NGP (lanes 7–9), and K562 (lanes 13–15) are all human cell lines and have differing levels of unlinkase activity; RRL (rabbit reticulocyte lysate) (lanes 10–12), contrary to previous publications, did not exhibit unlinkase activity in this assay (lanes 10–12).

Using our newly-developed assay, we wanted to determine if levels of unlinkase activity varied in extracts prepared from different human cell lines. We tested cytoplasmic extracts from human cells (Figure 5: HeLa, lanes 1–3, SK-OV-3, lanes 4–6, NGP, lanes 7–9, K562, lanes 13–15) using our W1-VPg31 radiolabeled substrate, and discovered that unlinkase activity varied among the human cell lines. The highest levels of unlinkase activity were observed in HeLa cell (Figure 5, lanes 1–3) and K562 cell (Figure 5, lanes 13–15) cytoplasmic extract. NGP cytoplasmic extract (Figure 5, lanes 7–9) exhibited lower levels of unlinkase activity than HeLa or K562 cells; cytoplasmic extract from SK-OV-3 cells (Figure 5, lanes 4–6) was observed to have the lowest levels of unlinkase activity of the different human cell lines examined. While levels of unlinkase activity differ, no correlation can be made between the levels of unlinkase activity and infectivity of the cell line. HeLa cells, with the highest levels of observed unlinkase activity, and NGP cells, which were observed to have a lower level of unlinkase activity, both support successful poliovirus infection [42]. In contrast, viral growth is somewhat limited in SK-OV-3 cells, which were observed to have low levels of unlinkase activity [43]; poliovirus or rhinovirus infection of K562 cells, which displayed high levels of unlinkase activity, results in a persistent infection [44].

Rabbit reticulocyte lysate (RRL), which has been reported to harbor unlinkase activity [3], [22], [24], was included as a control, but we were unable to detect unlinkase activity using our assay (Figure 5, lanes 10–12). This result was consistent over multiple experiments and did not change if the RRL was not treated with micrococcal nuclease prior to incubation with our radiolabeled substrate (data not shown). We conclude from these experiments that unlinkase activity varies in human cells but this variation in activity does not appear to be related to the ability of a host cell to support a successful picornaviral infection.

### Exogenous VPg does not affect unlinkase activity

**Figure 6:**
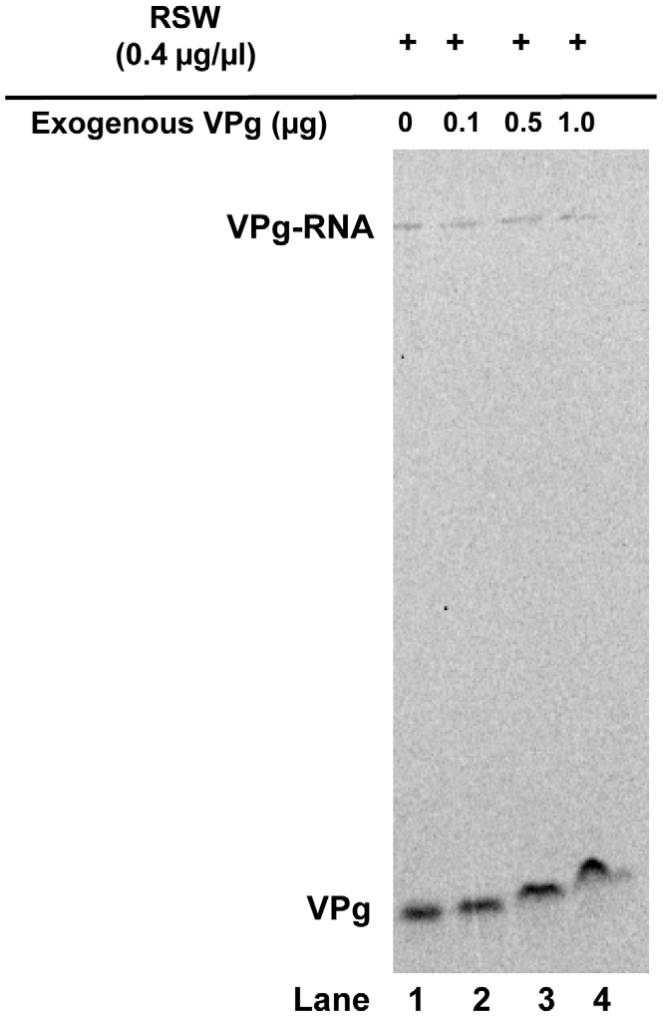
Exogenous poliovirus VPg does not affect unlinkase activity. Increasing amounts (0, 0.1, 0.5, and 1.0 µg) of exogenous synthetic poliovirus VPg peptide was added to full-length ^35^S-methionine-labeled substrate W1-VPg 31 virion RNA and RSW (0.4 µg/µl or 20 µg total) as an unlinkase source. Neither the addition of exogenous viral peptide (lanes 2–4) nor the addition of anti-VPg antibodies had an effect on unlinkase activity (data not shown).

Studies using proteinase K to cleave the VPg protein on picornavirus RNA, while leaving the tyrosyl-RNA phosphodiester bond intact, indicate that the length of the attached VPg is not important for unlinkase recognition [18], [19]. However, these experiments may not factor in VPg characteristics. VPg is a highly basic protein that likely associates with the negatively-charged backbone of the adjoining RNA molecule; it is possible that this interaction is masking the tyrosyl-RNA phosphodiester bond from unlinkase. In cleaving the majority of the connecting VPg, and therefore increasing exposure of the tyrosyl-RNA covalent bond, proteinase K may be enhancing unlinkase activity. Based on the unique characteristics of VPg and its potentially strong interaction with the adjoining picornavirus RNA, we hypothesized that there is a component of unlinkase that specifically recognizes and dissociates VPg from RNA in order to unmask the tyrosyl-RNA bond. To test this hypothesis, exogenous VPg was added as a potential competitor to reactions using RSW as an unlinkase source (Figure 6, compare lane 1 with lanes 2–4). We did not observe an effect on the efficiency of cleavage of the full-length radiolabeled RNA substrate, only a slower migration of the liberated radiolabeled VPg due to excess of the co-migrating unlabeled small viral protein (Figure 6, lanes 2–4). Additionally, antibodies specific for poliovirus VPg did not interfere with the unlinkase reaction (data not shown). We conclude that full-length VPg has no significant role in unlinkase activity.

### Unlinkase activity cleaves a human rhinovirus substrate with the same efficiency as a poliovirus substrate

**Figure 7:**
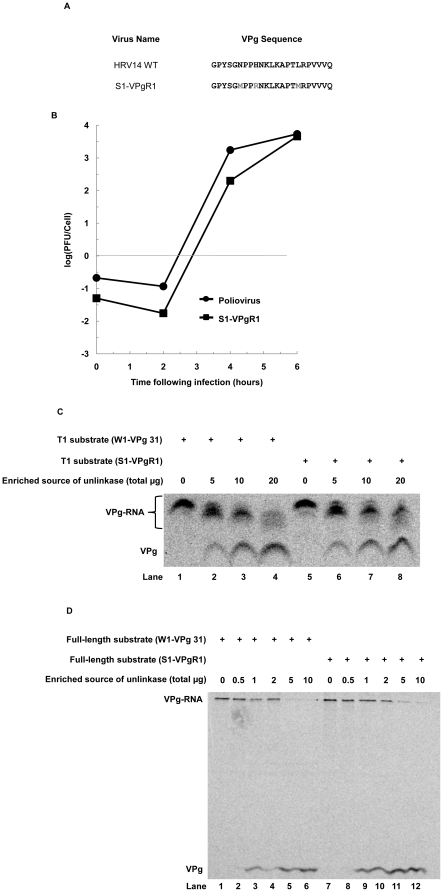
Unlinkase activity on a poliovirus versus a rhinovirus substrate. (A) VPg sequences of HRV14 and S1-VPgR1 (wild type poliovirus mutated to encode an HRV14 VPg that encodes two methionine residues). (B) Single-cycle growth assay of wild type poliovirus (closed circles) and S1-VPgR1 (closed squares). ^35^S-methionine radiolabeled W1-VPg 31 or S1-VPgR1 substrate that was treated with RNase T1 (C) or untreated (D) was incubated with increasing amounts of total protein from a partially-purified fraction of unlinkase activity (Fraction CA) ((C) 0, 5, 10, and 20 µg and (D) 0, 0.5, 1, 2, 5 and 10 µg) to demonstrate that unlinkase activity recognizes each substrate equally.

To determine if unlinkase activity can recognize and cleave a human rhinovirus substrate, we developed a chimeric virus consisting of a poliovirus genome that encodes a human rhinovirus 14 (HRV 14) VPg; the rhinovirus VPg was also mutated to encode two methionine residues for radiolabeling. We developed this chimeric virus, termed S1-VPgR1 (Figure 7A), due to the difficulty of generating sufficient amounts of purified virion RNA from human rhinovirus-infected HeLa cells. Growth kinetics determined that this virus chimera has a somewhat slower growth phenotype at four hours post infection when compared to wild type poliovirus in a single-cycle growth assay, but this difference is eliminated by six hours post infection, when virions are harvested for virus RNA purification (Figure 7B). S1-VPgR1 was used to infect HeLa cells in the presence of ^35^S-methionine, and virion RNA was purified. T1-treated virion RNA (Figure 7C) or full-length virion RNA (Figure 7D) from both W1-VPg31 and S1-VPgR1 radiolabeled substrates was incubated with an unlinkase source to determine if cleavage efficiency differed between the two viruses. RNase T1-generated substrates from both viruses (Figure 7C) were incubated with 0, 5, 10 or 20 µg of total protein from an enriched unlinkase activity source (Fraction CA), and no significant difference in cleavage efficiency between the two substrates was observed (Figure 7C compare lanes 4 and 8). Full-length substrates from both viruses (Figure 7D) were incubated with 0.5, 1, 2, 5, or 10 µg of total protein from the same enriched unlinkase activity source, and again no significant differences in cleavage between the two substrates were observed (Figure 7D compare lanes 6 and 12). No difference was observed for unlinkase cleavage of either substrate, leading to the conclusion that this enzyme can recognize and cleave a human rhinovirus substrate.

## Discussion

The linking of a protein to nucleic acid, ultimately resulting in a phosphodiester bond, is not a unique event, as evidenced in several virus families, prokaryotes, and eukaryotic cells. Enzymes that recognize and cleave this bond, however, are far more unusual. Such enzymes have been reported in prokaryotes, where proteins are regulated by uridylylation. Uridylyl-removing and uridylyltransferase enzymes are involved in prokaryotic regulation of glutamine synthetase [45] and galactose metabolism [46], [47]. To date, there have been two identified tyrosyl-DNA phosphodiesterases in eukaryotes: tyrosyl-DNA phosphodiesterase 1 (Tdp1) and tyrosyl-DNA phosphodiesterase 2 (formerly TTRAP), which primarily recognize and cleave 3′ and 5′ tyrosyl-DNA phosphodiester bonds, respectively [13], [14], [15], [16]. No RNA-tyrosyl phosphodiesterases have been identified; yet, there is overwhelming evidence that at least one such activity exists in mammalian cells as demonstrated by the removal of the 5′ terminal VPg from the genomic RNAs of picornaviruses. It might be assumed that one of the already-identified tyrosyl-DNA phosphodiesterases is also responsible for this tyrosyl-RNA phosphodiesterase activity, but this is not the case. We have determined that Tdp1 does not recognize and cleave a picornavirus tyrosyl-RNA substrate, nor does a source of unlinkase cleave a Tdp1 substrate (data not shown). Additionally, multiple mass spectrometry analyses have confirmed that neither Tdp1 nor TTRAP are found in any enriched unlinkase activity-contained protein preparations (data not shown). These findings are not surprising as it has been previously demonstrated that unlinkase recognizes and cleaves a very specific 5′ tyrosyl-RNA bond; it cannot cleave a synthetically generated 5′ tyrosyl-DNA bond [20], nor can it cleave a serine-RNA bond [21], [26].

Although the identity of unlinkase remains to be determined, its activity has been partially characterized. It is still unknown if this enzymatic activity is the result of a single protein, a multi-protein complex, or even possibly a ribonucleoprotein complex. We have confirmed that a longer length of the attached RNA to VPg increases the efficiency of the unlinkase activity. Why increasing the length of the genomic RNA increases the efficiency is currently unknown. It is possible that other sequences and/or structures in the picornavirus genome enhance recognition of the substrate bond, or that a longer RNA length is required for proper docking of the enzyme (potentially a complex). We explored the possibility of unlinkase activity resulting from the picornavirus genome acting as a ribozyme, but we could find no evidence to support this hypothesis (data not shown). Regardless of the mechanism, a host factor contributes to unlinkase activity and still must be identified.

We have determined that unlinkase can successfully recognize and cleave a picornaviral chimeric substrate that has a human rhinovirus 14 VPg covalently attached to the poliovirus genome. When accounting for the specific activity of the two picornavirus substrates, a protein fraction enriched for unlinkase activity had no preference for either the poliovirus or rhinovirus-poliovirus chimeric substrate, even though the amino acid sequence identity between the two VPg sequences is only 44%. Further supporting the conclusion that the covalently attached RNA is more important than the VPg protein is our data demonstrating that unlinkase activity remains unchanged in the presence of exogenous synthetic VPg as well as anti-VPg antibodies. The exogenous VPg was unable to sequester the unlinkase activity, leading to the conclusion that VPg alone, the product of this reaction, does not interact with the component(s) of the activity.

We wanted to determine if the level of unlinkase activity in extracts derived from different human cell lines was altered when compared to HeLa cell extract. Depending on the cell line tested, it was determined that activity did vary. K562, a human hematopoietic cell line, has unlinkase activity levels comparable to those in HeLa cells. Cytoplasmic extracts from SK-OV-3 cells, an ovarian carcinoma cell line, and NGP cells, a neuroblastoma cell line, had significantly less unlinkase activity compared to HeLa cells. It is noteworthy that unlinkase activity levels cannot be correlated to picornavirus infectivity; the significance of the altered levels of unlinkase activity in the cell lines tested remains to be determined. As a control, we also tested activity in rabbit reticulocyte lysate (RRL). Multiple groups have reported the presence of unlinkase activity in RRL, yet we have been unable to repeat these results using our assay and conditions. Our work is not the first indication that RRL has limited unlinkase activity during short incubation times [24], [48]. It is possible that significantly more protein from RRL than from human cell extract is necessary to achieve cleavage of the viral substrate (a plausible explanation as partial purification of the enzyme using RRL as a source has not been reported) or that a much longer incubation time is needed to observe cleavage of the substrate.

We explored the possibility that unlinkase activity might vary over the course of a picornavirus infection of HeLa cells. Our results were somewhat surprising, as one might expect unlinkase activity to decrease as the virus assumes control over the cellular transcription and translational machinery. In addition to the expectation that protein(s) responsible for unlinkase activity might be degraded and not replenished, we also considered the possibility that the activity would decrease as the infection progressed, providing more viral substrate and leaving less unlinkase available to act upon exogenous radiolabeled substrate. However, this was not what was observed, as unlinkase activity remained constant throughout the course of poliovirus infection. It is possible that unlinkase levels do not change over the course of the infection, but that picornavirus genomic RNA may be unavailable to unlinkase activity due to association with membranous vesicles in replication complexes, or through an association with viral capsid proteins.

The rationale for why unlinkase activity remains unchanged over the course of a typical poliovirus infection may offer insight into the role of unlinkase activity in the replication cycle of the virus. We hypothesize that unlinkase activity is usurped by the virus to distinguish templates for translation versus those destined to become replication templates or to be encapsidated. If unlinkase activity is used for this purpose, this mechanism may only be required early in the infection when it is crucial that templates are available for viral protein synthesis. It has been demonstrated that poliovirus genomes undergo a successful round of translation before being utilized as a template for replication [49], [50]. It is currently unknown if unlinkase cleaves VPg from the genomic RNA before or after the initiation of translation, but it has been reported that ribosomes can associate with full-length VPg-RNA, although this study used RRL as a source of translation extract [48]. RRL was later shown to produce aberrant protein products when programmed with poliovirus virion RNA [51] and is suspected to be deficient in unlinkase activity (Figure 5, lanes 10-12). VPg removal from viral RNA could conceivably be a marker to distinguish a template that will maintain, at least initially, sustained levels of viral protein synthesis and RNA replication. Such efficiency may be more important prior to the peak of RNA replication, when viral proteins must carry out the initial rounds of replication of the picornavirus genome. Once a basal threshold of viral proteins sufficient for maximal viral RNA synthesis is produced, it may not be crucial to maintain efficient translation of viral proteins. An explanation for how picornaviruses protect RNA replication templates and full-length genomes for encapsidation from VPg removal from virion RNA is via coupling RNA replication and encapsidation, as these viral processes have been demonstrated to be coupled *in vitro* [32], [50], [52], [53], [54]; it is feasible that the capsid proteins shield the nascent viral genomic RNA from unlinkase activity.

## References

[1] Wimmer E (1982). Genome-linked proteins of viruses.. Cell.

[2] Drygin YF (1998). Natural covalent complexes of nucleic acids and proteins: some comments on practice and theory on the path from well-known complexes to new ones.. Nucleic Acids Res.

[3] Pettersson RF, Ambros V, Baltimore D (1978). Identification of a protein linked to nascent poliovirus RNA and to the polyuridylic acid of negative-strand RNA.. J Virol.

[4] Rothberg PG, Harris TJ, Nomoto A, Wimmer E (1978). O4-(5′-uridylyl)tyrosine is the bond between the genome-linked protein and the RNA of poliovirus.. Proc Natl Acad Sci U S A.

[5] Flanegan JB, Pettersson RF, Ambros V, Hewlett NJ, Baltimore D (1977). Covalent linkage of a protein to a defined nucleotide sequence at the 5′-terminus of virion and replicative intermediate RNAs of poliovirus.. Proc Natl Acad Sci U S A.

[6] Lee YF, Nomoto A, Detjen BM, Wimmer E (1977). A protein covalently linked to poliovirus genome RNA.. Proc Natl Acad Sci U S A.

[7] Kitamura N, Semler BL, Rothberg PG, Larsen GR, Adler CJ (1981). Primary structure, gene organization and polypeptide expression of poliovirus RNA.. Nature.

[8] Nomoto A, Kitamura N, Golini F, Wimmer E (1977). The 5′-terminal structures of poliovirion RNA and poliovirus mRNA differ only in the genome-linked protein VPg.. Proc Natl Acad Sci U S A.

[9] Nomoto A, Detjen B, Pozzatti R, Wimmer E (1977). The location of the polio genome protein in viral RNAs and its implication for RNA synthesis.. Nature.

[10] Paul AV, van Boom JH, Filippov D, Wimmer E (1998). Protein-primed RNA synthesis by purified poliovirus RNA polymerase.. Nature.

[11] Toyoda H, Yang CF, Takeda N, Nomoto A, Wimmer E (1987). Analysis of RNA synthesis of type 1 poliovirus by using an in vitro molecular genetic approach.. J Virol.

[12] Osheroff N (1989). Biochemical basis for the interactions of type I and type II topoisomerases with DNA.. Pharmacol Ther.

[13] Cortes Ledesma F, El Khamisy SF, Zuma MC, Osborn K, Caldecott KW (2009). A human 5′-tyrosyl DNA phosphodiesterase that repairs topoisomerase-mediated DNA damage.. Nature.

[14] Pouliot JJ, Yao KC, Robertson CA, Nash HA (1999). Yeast gene for a Tyr-DNA phosphodiesterase that repairs topoisomerase I complexes.. Science.

[15] Interthal H, Pouliot JJ, Champoux JJ (2001). The tyrosyl-DNA phosphodiesterase Tdp1 is a member of the phospholipase D superfamily.. Proc Natl Acad Sci U S A.

[16] Yang SW, Burgin AB, Huizenga BN, Robertson CA, Yao KC (1996). A eukaryotic enzyme that can disjoin dead-end covalent complexes between DNA and type I topoisomerases.. Proc Natl Acad Sci U S A.

[17] Ambros V, Pettersson RF, Baltimore D (1978). An enzymatic activity in uninfected cells that cleaves the linkage between poliovirion RNA and the 5′ terminal protein.. Cell.

[18] Ambros V, Baltimore D (1980). Purification and properties of a HeLa cell enzyme able to remove the 5′-terminal protein from poliovirus RNA.. J Biol Chem.

[19] Gulevich AY, Yusupova RA, Drygin YF (2002). VPg unlinkase, the phosphodiesterase that hydrolyzes the bond between VPg and picornavirus RNA: a minimal nucleic moiety of the substrate.. Biochemistry (Moscow).

[20] Shabanov AA, Kaliuzhnyi AA, Gottikh MB, Drygin YF (1996). Natural substrates of uridylylpolynucleotide-(5′P--->O)-tyrosine phosphodiesterase–an enzyme, hydrolyzing the covalent bond between RNA and picornaviral VPg and a synthetic model of them.. Biochemistry (Moscow).

[21] Drygin YF, Shabanov AA, Bogdanov AA (1988). An enzyme which specifically splits a covalent bond between picornaviral RNA and VPg.. FEBS Lett.

[22] Sangar DV, Bryant J, Harris TJ, Brown F, Rowlands DJ (1981). Removal of the genome-linked protein of foot-and-mouth disease virus by rabbit reticulocyte lysate.. J Virol.

[23] Ambros V, Baltimore D (1978). Protein is linked to the 5′ end of poliovirus RNA by a phosphodiester linkage to tyrosine.. J Biol Chem.

[24] Dorner AJ, Rothberg PG, Wimmer E (1981). The fate of VPg during in vitro translation of poliovirus RNA.. FEBS Lett.

[25] Drygin Yu F, Siyanova E (1986). [Characteristics of an enzyme hydrolyzing the covalent bond between RNA and protein VPg of the encephalomyocarditis virus].. Biokhimia.

[26] De Varennes A, Lomonosoff GP, Shanks M, Maule AJ (1986). The stability of cowpea mosaic virus VPg in reticulocyte lysates.. J Gen Virol.

[27] Nomoto A, Lee YF, Wimmer E (1976). The 5′ end of poliovirus mRNA is not capped with m7G(5′)ppp(5′)Np.. Proc Natl Acad Sci U S A.

[28] Hewlett MJ, Rose JK, Baltimore D (1976). 5′-terminal structure of poliovirus polyribosomal RNA is pUp.. Proc Natl Acad Sci U S A.

[29] Pettersson RF, Flanegan JB, Rose JK, Baltimore D (1977). 5′-Terminal nucleotide sequences of polio virus polyribosomal RNA and virion RNA are identical.. Nature.

[30] Fernandez-Munoz R, Darnell JE (1976). Structural difference between the 5′ termini of viral and cellular mRNA in poliovirus-infected cells: possible basis for the inhibition of host protein synthesis.. J Virol.

[31] Fernandez-Munoz R, Lavi U (1977). 5′ termini of poliovirus RNA: difference between virion and nonencapsidated 35S RNA.. J Virol.

[32] Baltimore D, Girard M, Darnell JE (1966). Aspects of the synthesis of poliovirus RNA and the formation of virus particles.. Virology.

[33] Puck TT, Marcus PI, Cieciura SJ (1956). Clonal growth of mammalian cells in vitro; growth characteristics of colonies from single HeLa cells with and without a feeder layer.. J Exp Med.

[34] Lozzio CB, Lozzio BB (1973). Cytotoxicity of a factor isolated from human spleen.. J Natl Cancer Inst.

[35] Brodeur GM, Sekhon G, Goldstein MN (1977). Chromosomal aberrations in human neuroblastomas.. Cancer.

[36] Dildine SL, Semler BL (1992). Conservation of RNA-protein interactions among picornaviruses.. J Virol.

[37] Walter BL, Parsley TB, Ehrenfeld E, Semler BL (2002). Distinct poly(rC) binding protein KH domain determinants for poliovirus translation initiation and viral RNA replication.. J Virol.

[38] Kuhn RJ, Tada H, Ypma-Wong MF, Dunn JJ, Semler BL (1988). Construction of a “mutagenesis cartridge” for poliovirus genome-linked viral protein: isolation and characterization of viable and nonviable mutants.. Proc Natl Acad Sci U S A.

[39] Kuhn RJ, Tada H, Ypma-Wong MF, Semler BL, Wimmer E (1988). Mutational analysis of the genome-linked protein VPg of poliovirus.. J Virol.

[40] Cheney IW, Naim S, Shim JH, Reinhardt M, Pai B (2003). Viability of poliovirus/rhinovirus VPg chimeric viruses and identification of an amino acid residue in the VPg gene critical for viral RNA replication.. J Virol.

[41] Doersen CJ, Stanbridge EJ (1979). Cytoplasmic inheritance of erythromycin resistance in human cells.. Proc Natl Acad Sci U S A.

[42] Haller AA, Stewart SR, Semler BL (1996). Attenuation stem-loop lesions in the 5′ noncoding region of poliovirus RNA: neuronal cell-specific translation defects.. J Virol.

[43] Brunner JE, Ertel KJ, Rozovics JM, Semler BL (2010). Delayed kinetics of poliovirus RNA synthesis in a human cell line with reduced levels of hnRNP C proteins.. Virology.

[44] Lloyd RE, Bovee M (1993). Persistent infection of human erythroblastoid cells by poliovirus.. Virology.

[45] Adler SP, Purich D, Stadtman ER (1975). Cascade control of Escherichia coli glutamine synthetase. Properties of the PII regulatory protein and the uridylyltransferase-uridylyl-removing enzyme.. J Biol Chem.

[46] Arabshahi A, Brody RS, Smallwood A, Tsai TC, Frey PA (1986). Galactose-1-phosphate uridylyltransferase. Purification of the enzyme and stereochemical course of each step of the double-displacement mechanism.. Biochemistry.

[47] Geeganage S, Frey PA (1998). Transient kinetics of formation and reaction of the uridylyl-enzyme form of galactose-1-P uridylyltransferase and its Q168R-variant: insight into the molecular basis of galactosemia.. Biochemistry.

[48] Golini F, Semler BL, Dorner AJ, Wimmer E (1980). Protein-linked RNA of poliovirus is competent to form an initiation complex of translation in vitro.. Nature.

[49] Novak JE, Kirkegaard K (1994). Coupling between genome translation and replication in an RNA virus.. Genes Dev.

[50] Nugent CI, Johnson KL, Sarnow P, Kirkegaard K (1999). Functional coupling between replication and packaging of poliovirus replicon RNA.. J Virol.

[51] Dorner AJ, Semler BL, Jackson RJ, Hanecak R, Duprey E (1984). In vitro translation of poliovirus RNA: utilization of internal initiation sites in reticulocyte lysate.. J Virol.

[52] Caliguiri LA, Compans RW (1973). The formation of poliovirus particles in association with the RNA replication complexes.. J Gen Virol.

[53] Pfister T, Pasamontes L, Troxler M, Egger D, Bienz K (1992). Immunocytochemical localization of capsid-related particles in subcellular fractions of poliovirus-infected cells.. Virology.

[54] Liu Y, Wang C, Mueller S, Paul AV, Wimmer E (2010). Direct Interaction between Two Viral Proteins, the Nonstructural Protein 2CATPase and the Capsid Protein VP3, Is Required for Enterovirus Morphogenesis.. PLoS Pathog.

